# Optimizing the Implementation of Clinical Predictive Models to Minimize National Costs: Sepsis Case Study

**DOI:** 10.2196/43486

**Published:** 2023-02-13

**Authors:** Parker Rogers, Aaron E Boussina, Supreeth P Shashikumar, Gabriel Wardi, Christopher A Longhurst, Shamim Nemati

**Affiliations:** 1 Department of Economics University of California, San Diego La Jolla, CA United States; 2 Department of Biomedical Informatics University of California, San Diego La Jolla, CA United States; 3 Department of Emergency Medicine University of California, San Diego La Jolla, CA United States; 4 Division of Pulmonary, Critical Care and Sleep Medicine University of California, San Diego La Jolla, CA United States

**Keywords:** sepsis, machine learning, evaluation, utility assessment, workflow simulation, simulation, model, implementation, data, acute kidney injury, injury, technology, care, diagnosis, clinical, cost

## Abstract

**Background:**

Sepsis costs and incidence vary dramatically across diagnostic categories, warranting a customized approach for implementing predictive models.

**Objective:**

The aim of this study was to optimize the parameters of a sepsis prediction model within distinct patient groups to minimize the excess cost of sepsis care and analyze the potential effect of factors contributing to end-user response to sepsis alerts on overall model utility.

**Methods:**

We calculated the excess costs of sepsis to the Centers for Medicare and Medicaid Services (CMS) by comparing patients with and without a secondary sepsis diagnosis but with the same primary diagnosis and baseline comorbidities. We optimized the parameters of a sepsis prediction algorithm across different diagnostic categories to minimize these excess costs. At the optima, we evaluated diagnostic odds ratios and analyzed the impact of compliance factors such as noncompliance, treatment efficacy, and tolerance for false alarms on the net benefit of triggering sepsis alerts.

**Results:**

Compliance factors significantly contributed to the net benefit of triggering a sepsis alert. However, a customized deployment policy can achieve a significantly higher diagnostic odds ratio and reduced costs of sepsis care. Implementing our optimization routine with powerful predictive models could result in US $4.6 billion in excess cost savings for CMS.

**Conclusions:**

We designed a framework for customizing sepsis alert protocols within different diagnostic categories to minimize excess costs and analyzed model performance as a function of false alarm tolerance and compliance with model recommendations. We provide a framework that CMS policymakers could use to recommend minimum adherence rates to the early recognition and appropriate care of sepsis that is sensitive to hospital department-level incidence rates and national excess costs. Customizing the implementation of clinical predictive models by accounting for various behavioral and economic factors may improve the practical benefit of predictive models.

## Introduction

Recent advancements in machine learning (ML) and the proliferation of health care data have led to widespread excitement about using these technologies to improve care [1,2]. Predictive analytic models in domains such as sepsis [3-5], acute kidney injury [6], respiratory failure [7], and general deterioration [8] have been proposed to improve the timely administration of lifesaving treatments and mitigate expensive downstream complications. It has been argued that a more tailored approach that accounts for implementation constraints that may differ across care settings can further enhance the adoption of such systems [9].

Despite its importance, the process of implementing predictive analytics solutions has received little attention relative to the development of the underlying ML models [10]. Algorithms are becoming more sophisticated, and the infrastructure that allows real-time interoperable deployment of predictive analytics solutions is expanding [11,12]. This increase in potential and complexity underscores the practical importance of understanding the implementation policy layer, which captures the clinical workflow, response protocols, and operational constraints. Notably, the dominant evaluation methods within the ML community, such as the area under the receiver operating characteristic curve, often do not consider the effect of this policy layer on model performance [13]. Moreover, such performance metrics do not consider the user response to prediction and the effectiveness of the treatment protocols [14]. However, the operational constraints can often go beyond behavioral factors and may encompass quality improvement mandates and cost-saving objectives [15].

This work focuses on the management of sepsis—a common and lethal condition caused by a dysregulated host response to infection [16]—although our framework can be applied to other hospital-acquired conditions [17]. Sepsis afflicts over 49 million people worldwide and accounts for over 11 million deaths per year [18]. In 2018, the US Medicare program (including fee-for-service and Medicare Advantage) incurred US $41.5 billion in sepsis-related inpatient hospital admissions and skilled nursing facility care costs [19].

We propose a framework for improving the implementation of ML-based electronic health record alerts. Our framework aims to minimize the costs of sepsis to payers, which are potentially avoidable through early detection, timely administration of antibiotics, and prevention of overtreatment (ie, excess costs) [5,20,21]. Importantly, these costs can differ by diagnostic category (and by extension, hospital departments) due to differences in incidence rates, patient susceptibility, and physician adherence. Thus, an additional contribution of this work is our estimation of the excess costs of sepsis at the diagnostic-category and national level, that is, the costs paid by the Centers for Medicare and Medicaid Services (CMS). Our optimization framework uses these cost estimates and selects specific decision thresholds for each diagnostic category, differing from other cost-benefit frameworks that set decision thresholds uniformly [22,23]. We simulate how thresholds and model outcomes can crucially depend on physician adherence and sepsis incidence rates. In summary, we provide a framework that CMS policymakers could use to recommend minimum adherence rates to the early recognition and appropriate care of sepsis that is sensitive to hospital department-level incidence rates and national excess costs. This tailored approach results in higher cost savings and diagnostic accuracy.

## Methods

**Figure 1:**
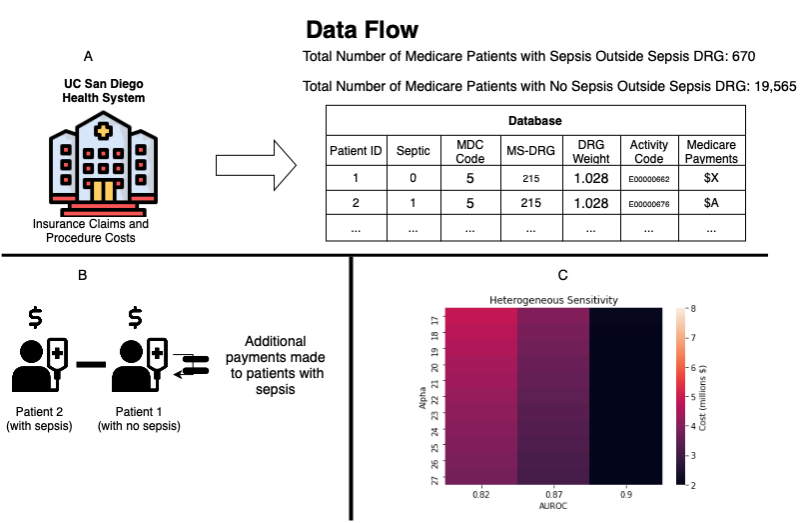
Overall framework for assessment of attributable cost to sepsis and optimization of predictive model parameters. (A) Data collection, (B) Data manipulation, (C) Minimizing additional costs from sepsis by choosing sensitivity/specificity pairs across departments. AUROC: area under the receiver operating characteristic curve; DRG: diagnosis-related group; MDC: major diagnostic category; MS-DRG: Medicare Severity Diagnosis-Related Group; UC: University of California.

We conducted a retrospective observational study with the following 3 broad steps: data collection, excess cost estimation, and cost minimization (Figure 1). This was done in accordance with STROBE (Strengthening the Reporting of Observational Studies in Epidemiology) guidelines [24].

### Ethics Approval

The use of deidentified data utilized in this study was approved by the institutional review board of University of California (UC) San Diego (approval 800257). The requirement for informed consent was waived by the institutional review board committee, as the use of deidentified retrospective data does not require patient consent under the Health Insurance Portability and Accountability Act privacy regulations.

### Data Sets and Definitions

We collected Medicare claims data from patients 18 years or older at UC San Diego Health (UCSDH), an academic health system, between October 2016 and July 2020. These data included the following necessary components: (1) the Medicare Severity Diagnosis-Related Groups (DRGs) [25] diagnosis code for each patient and their corresponding DRG weights, (2) the total amount paid by Medicare for the patient, and (3) the Charlson comorbidity index of the patient upon admission. We included patients with International Classification of Diseases-Tenth Revision (ICD-10) codes for severe sepsis (ICD-9: 99592 and ICD-10: R6520) and septic shock (ICD-9: 78552 and ICD-10: R6521). We selected these because of their inclusion in the CMS Quality Measure for Severe Sepsis and Septic Shock, which has impacted sepsis care across the United States and provides a standardized approach to management [26]. Throughout this paper, the term “sepsis” refers to the definitions of severe sepsis and septic shock. The major diagnostic categories (MDCs) are formed by dividing all possible principal diagnoses into 25 mutually exclusive diagnosis areas, which roughly correspond to hospital departments.

### Excess Cost of Sepsis

Our efforts to quantify the costs of missed diagnoses (ie, false negatives) provide a new estimate of the avoidable costs of severe sepsis and septic shock across broad diagnostic categories. To quantify, we used granular insurance claims data under the Medicare prospective payment system. We focused on hospitalized Medicare patients, as payments are specific to DRGs—a payment classification system determined mainly by the diagnosis that caused a patient to become hospitalized. This system groups clinically similar conditions that require similar levels of inpatient resources. This categorization also allows us to show the public value of our optimization routine. We excluded patients from sepsis-related DRGs (870, 871, 872) from our analysis because our objective was to assess the *excess inpatient cost* of sepsis for other DRGs. As such, we gathered all patients with severe sepsis and septic shock in nonsepsis DRGs and a group of control patients in those same DRGs. This strategy allowed a cost comparison between individuals with similar primary diagnoses (ie, underlying conditions) but different secondary sepsis diagnoses. These data included 670 patients diagnosed with severe sepsis and septic shock across 131 DRGs and 19,565 control group patients.

We adjusted for other underlying factors that drive cost differences between patients with sepsis and no sepsis by matching a comparison individual to each patient with sepsis [27]. For each patient with sepsis, this matching procedure selected from all comparison individuals within the same DRG code/weight the patient with the most similar Charlson comorbidity index to the given patient with sepsis. This matching procedure does not guarantee conditional independence (ie, causality between sepsis and excess costs), but we use it to approximate excess costs for the sake of simulation. Further, limiting the selection to patients within the same DRG weight accounts for changes in DRG payments over time. With sets of patients with sepsis matched to control patients, we subtracted the Medicare payments made for the matched patients from the payments made for the patients with sepsis. This difference represents the excess costs that Medicare paid for sepsis above the underlying costs attributable to the primary patient diagnosis.

We repeated this procedure for all patients with sepsis to form a distribution of excess costs across DRGs. We then averaged these DRG-specific excess cost estimates by MDC, which comprises 16 mutually exclusive diagnosis areas within our data set [28]. We show that the added costs from sepsis diagnoses vary dramatically by these diagnostic categories and, by extension, different hospital departments.

In an effort to calculate the national excess cost of sepsis, we then scaled our excess cost estimates to the national level to show the public impact of early detection and treatment (see Multimedia Appendix 1 for more details). To scale, we first multiplied UCSDH payments by the ratio of UCSDH payments to average US payments by DRG [29]. Then, we scaled the total patients treated at UCSDH to the national number of patients with sepsis by using the share of Medicare patients treated at UCSDH. Lastly, we aggregated the payments across all patients. We have validated this scaling approach, as shown in Multimedia Appendix 1, and we found that we closely estimated the total national inpatient sepsis costs documented in Medicare cost data [30] (among sepsis DRGs 870-872) by scaling UCSDH total sepsis costs to the national level.

### Modeling and Optimization

We used the sepsis prediction model by Shashikumar et al [5] to develop an optimization framework that chooses the model’s classification thresholds to minimize the additional costs from sepsis by the MDCs. In the context of sepsis prediction, classification thresholds determine above which probability the model tags a patient as septic. Although we optimized across diagnostic categories, our routine could also be implemented across hospital departments or, alternatively, across more granular patient subpopulations. The intuition behind the value of our implementation rests on the idea that diagnostic categories may determine whether patients with sepsis are costly relative to patients with no sepsis, which could potentially merit customized classification thresholds that account for these category-specific nuances. Additionally, departments could have different rates of sepsis, which may require different thresholds to avoid a large number of missed detections. By allowing algorithmic sensitivity to adjust to these idiosyncrasies, ML algorithms may further reduce costs. Our optimizer is constrained by the predictive model’s area under the curve (AUC): as the optimizer chooses a higher sensitivity to sepsis to reduce the costs of sepsis, the specificity of the model decreases, increasing the false alarm rate. As noted above, false alarms can also be costly: treating patients with broad-spectrum antibiotics can cause adverse effects and is expensive. Thus, the algorithm must balance the trade-off between the cost of undertreatment and overtreatment.

Let *FN_i_* represent the number of false negatives (ie, the missed cases of sepsis) within a given MDC category, and let Cost_*FN_i_* represent the cost of missing sepsis within this category. The miss rate (ie, *1 – sens_i_*) is a quantity that depends on the selection of the risk score threshold within the given MDC category *i*. Furthermore, let the estimated functional form *f( )* provide a mapping from the chosen sensitivity to the false positive rate (or false alarm rate). Note that this function is constrained by the model AUC and reflects the balance of model sensitivity and false alarms (see fifth point in Multimedia Appendix 1). This function may also vary by MDC, but for simplicity, we use a common functional form *f( )*. The optimization routine is given by the following formula:

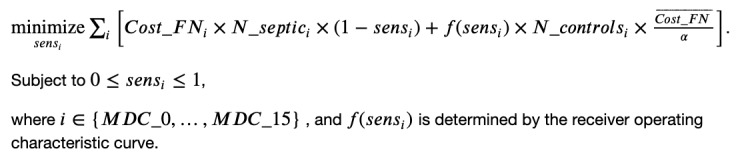



Notice that the algorithm chooses sensitivity values (ie, *sens_i_*) across 16 broad diagnostic categories (ie, *i*) to minimize costs. The left-hand side of the objective function captures the excess costs or the cost of false negatives: the MDC’s average cost of false negatives multiplied by the number of patients with sepsis in the diagnostic category (ie, *N_septic_i_*) multiplied by the miss rate. The right-hand side of the objective function captures the costs of false positives: the false alarm rate multiplied by the number of patients who are not septic in the MDC (ie, *N_controls_i_*) multiplied by the average costs of false negatives divided by the conversion factor *α.* This conversion factor is a variable that maps the cost of false positives to the cost of false negatives, as there may be costs of overtreatment (eg, administering antibiotics if patients do not have sepsis). Our simulations, as detailed below, consider various levels of *α*, allowing for comparisons across different parameter assumptions. We also included parameters in the model that characterize physician adherence to sepsis alarms and tolerances to false alarms (ie, overtreatment). For simplicity, our model implicitly assumes that most sepsis costs are associated with the downstream consequences of sepsis, such as organ failure, need for intensive care, and prolonged hospitalization [15]. As such, we assume the costs of broad-spectrum antibiotics and other early sepsis treatments are negligible and thus excluded from our analysis [31].

### Simulations

We simulate a series of outcomes by implementing sepsis prediction algorithms with flexible classification thresholds. Simulation parameters and definitions are provided in Table 1. These parameters constitute several factors in the policy layer of ML algorithm implementation and may vary across hospitals and departments. There are potentially other factors when implementing an algorithm that will affect outcomes (eg, the effectiveness of treatment), but we focus on a few salient factors that have arisen, namely, antimicrobial resistance and issues stemming not from imperfect algorithms but from imperfect adherence to alarm triggers.

**Table 1:** Simulation parameters.

Parameter	Description	Range
Cost of false alarms (α)	The costs associated with overtreating sepsis (eg, costs of antibiotics, patient side effects)	17-27
Physician adherence (γ)	The rate at which physicians comply with the algorithm recommendations to treat	0.5-1.0
Area under the curve	The area under the receiver operating characteristic curve, which is a commonly used measure of predictive accuracy	0.82, 0.87, 0.9
Diagnostic odds ratio	Odds of a positive test in patients with disease relative to the odds of a positive test in patients without disease	10-120

#### Model Performance and False Alarm Tolerance

The first simulation illustrates the cost savings generated when choosing classification thresholds across diagnostic categories. The simulation presents cost savings achieved when using 3 different artificial intelligence models [5] with various levels of predictive performance (ie, receiver operating characteristic curve and AUC). These 3 artificial intelligence models are taken from the literature, and we apply them “out-of-the-box” by feeding them into our optimization framework. Each model’s receiver operating characteristic curve determines the levels of sensitivity and specificity that the algorithm can achieve and determines the trade-offs between underdiagnosis and overdiagnosis. We also simulated excess cost outcomes over a range of different tolerances to false alarms (eg, higher tolerance means that the costs of overtreatment are lower). This exercise illustrates the returns of allowing flexible classification thresholds across diagnostic categories for various ML algorithms and cost assumptions. We then calculated and presented the diagnostic odds ratios (DORs) at each accuracy level and cost assumption, given the optimized classification thresholds.

#### Physician Adherence

We then reformulated the optimizer to account for physician adherence. For a given classification threshold, low adherence leads to a lower detection rate as alarms are ignored. To illustrate the effects of physician adherence on costs, we ran a similar simulation to the above, but rather than considering 3 models of differing accuracy, we varied the adherence rate. Hence, the simulation calculated excess costs at the set of optima for different adherence parameters and costs of false alarms. Lower levels of γ indicate a lower level of physician adherence (see equation S2 in Multimedia Appendix 1).

#### Comparison to the Uniform Classification Threshold Chosen by Optimizer

We underscored the gains from optimizing classification thresholds by department. To this end, we did the same set of simulations when allowing only 1 classification threshold across departments. We then calculated the excess costs for different false alarm costs and accuracy levels at the optimal threshold. We also calculated the DORs at these optima.

#### Comparison to the Uniform Classification Threshold

We calculated the excess costs if the algorithm implementers use a uniform 80% sensitivity, representing a clinically useful target detection rate [5]. We calculated excess costs at different false alarm costs, physician adherence, and accuracy levels, and we calculated the DORs at the optima.

## Results

### Calculation of Excess Costs of Sepsis

**Figure 2:**
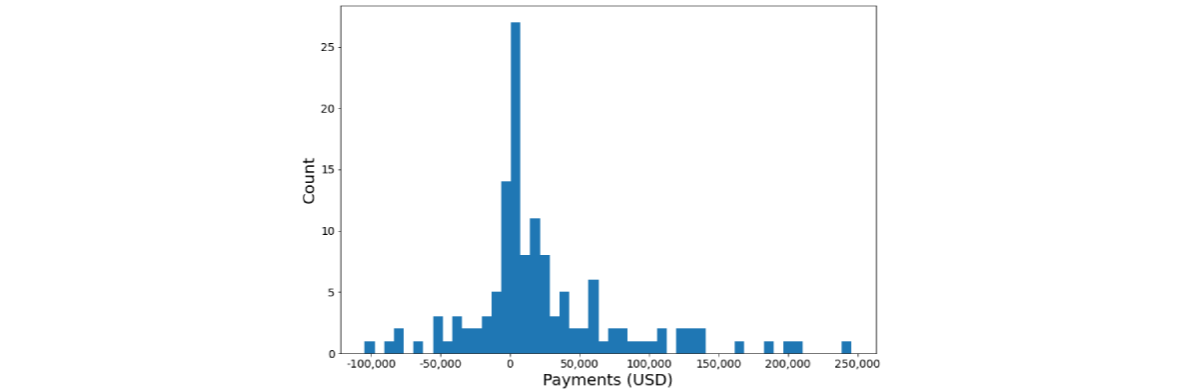
Distribution of mean excess sepsis payments over all diagnosis-related groups. This is the distribution of excess costs, as presented in Figure 3, but limited to the University of California San Diego Health cohort.

Figure 2 shows the distribution of mean excess inpatient sepsis payments by DRG. The distribution’s mean is US $23,929, and its median is US $8124. Importantly, this implies that, on average, patients with sepsis generate US $24,000 more charges than patients who are not septic within the same DRG (matched on baseline severity). Differences between payments for patients within the same DRG weight exist because Medicare reimburses extra for costlier hospital encounters. Patients with high cost-to-charge ratios receive additional payments to compensate for hospital losses, called as outlier payments [29]. Thus, if the costs of sepsis treatment or other nonsepsis treatments exceed a certain threshold, Medicare compensates the hospital a certain percentage of the costs above the standard Medicare payment. Hence, outlier payments drive the difference in Medicare payments within the same DRG weight. Notice that outlier payments also explain why some patients with sepsis are less costlier than patients with no sepsis: outlier payments for these patients with no sepsis happen to be higher for other care unrelated to sepsis. DRGs in the left tail of the distribution are those in the “Infectious and Parasitic Diseases, Systemic or Unspecified Sites” MDC code, which suggests that sepsis may lead to better treatment outcomes or, conversely, sepsis may hasten hospital discharge through death. By contrast, DRGs in the “Pre-MDC” seem to trigger outlier payments that are quite large relative to patients with no sepsis. The aggregated excess costs by MDC category are presented in Table S1 of Multimedia Appendix 1, which shows that sepsis can additionally cost Medicare up to US $85,000 per patient.

**Figure 3:**
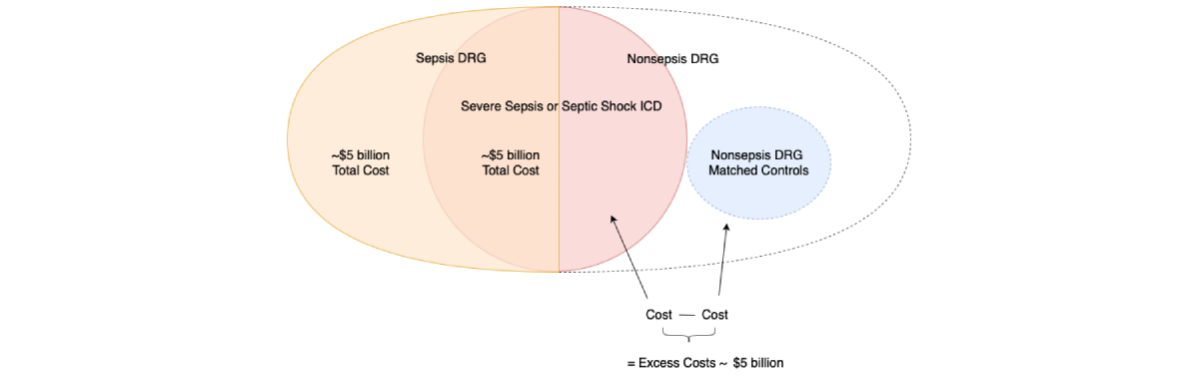
Venn diagram of the Medicare inpatient population (2016-2019) by diagnosis-related group and severe sepsis diagnosis. The US $5 billion total costs of severe sepsis and sepsis diagnosis-related groups with severe sepsis or septic shock International Classification of Diseases codes were calculated using University of California San Diego Health Medicare claims data and scaled to the national level. Note that these are total costs rather than excess costs. DRG: diagnosis-related group; ICD: International Classification of Diseases.

The second set of results describe the outcomes of a simulation of excess cost savings and DORs achieved by ML algorithms with fine-tuned classification thresholds. We estimate that the excess cost for inpatient sepsis cases in the United States is US $5.2 billion per year before predictive analytics implementation (see Multimedia Appendix 1 for details). Note that this estimate does not consider those patients whose primary diagnostic category is sepsis. Rather, it includes those who belong to nonsepsis DRGs who have secondary sepsis diagnoses. The former group incurs a total cost of roughly the same amount as the excess costs associated with our study’s patient cohort (see Figure 3). Additionally, our excess cost estimate ignores excess utilization of inpatient providers, skilled nursing facilities, and costs incurred due to the high 30-day sepsis readmission rates, which is estimated to be 20% [32,33]. Thus, our estimate likely provides a lower bound on excess costs.

Our simulation results show the savings achieved with our implementation across various assumptions. Our first set of results describes 3 simulation routines that differ by the degrees of freedom with which classification thresholds are chosen: (1) corresponding to 80% sensitivity, (2) uniform across all diagnostic categories, and (3) distinct and optimized for each diagnostic category. We present cost savings for each degree of freedom across various assumptions on ML accuracy and false-positive costs. Our second set of results provides the DORs for degrees of freedom (1-3) at the optima chosen to minimize costs across the same ML accuracy and false-positive cost assumptions.

### Cost Savings

#### Uniform Classification Threshold

**Figure 4:**
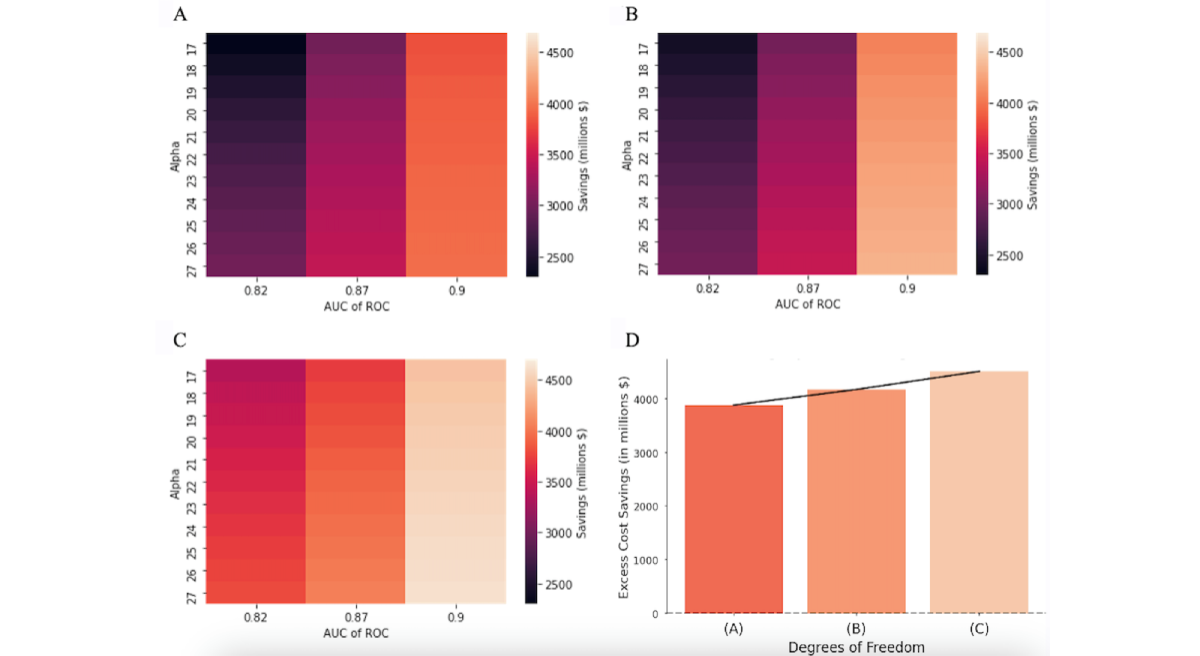
(A) Model cost savings when threshold is 80% sensitivity across departments. (B) Model cost savings when one threshold is chosen, but it is the same across departments. (C) Model cost savings when threshold is chosen across departments. (D) Model cost savings by each model type (A), (B), and (C). The cost savings estimates in (D) are for area under the curve=0.9 and α=20 across the 3 models. We choose α=20 to align with the maximum penalty for false alarms in the PhysioNet challenge [4]. AUC: area under the curve; ROC: receiver operating characteristic curve.

The first results detail cost savings when using a uniform recommendation of 80% sensitivity and applying it throughout the hospital at different false-positive costs and various levels of ML accuracy. Note, this implementation differs from the other two as the threshold is not optimized. Figure 4A shows that as the cost of false positives decreases (ie, higher α values), classification thresholds are chosen more aggressively, which leads to higher cost savings as more patients with sepsis are diagnosed and treated. Similarly, as the predictive power of the model increases (ie, higher AUC), savings increase. The most influential factor in cost savings is the model’s predictive power, with excess cost savings ranging from US $2.3 billion to US $3.9 billion.

#### Uniform Classification Threshold Chosen by Optimizer

Instead of relying on a uniform recommended threshold, implementers may choose one to minimize costs throughout the hospital. The simulation of this implementation shows to what extent cost savings would differ. Figure 4B shows that for every AUC-α pair, cost savings are higher when the threshold is chosen. Where savings are the highest, an optimized uniform threshold can save over US $400 million relative to the uniform recommended level (US $3.9 billion cost savings with 80% uniform and US $4.3 billion cost savings with uniform chosen). Cost savings exhibit similar patterns across α and AUC values as the above model.

#### Heterogenous Classification Thresholds Chosen by Optimizer

Further, implementers could optimize thresholds across broad diagnostic categories (or hospital departments). Figure 4C shows that the gains from choosing heterogenous thresholds by MDCs are the highest for lower-accuracy models. This discrepancy is illustrated by the difference between cost savings in the uniform model versus the heterogeneous model, with cost savings at AUC of 0.82 as high as US $3.7 billion when using heterogeneous thresholds compared to US $3 billion with the uniform model. At the pair where the highest savings are achieved, heterogeneous thresholds can save over US $300 million relative to uniform thresholds (US $4.3 billion cost savings with uniform and US $4.6 billion with heterogeneous) and almost US $700 million compared to the 80% standard. Cost savings exhibit similar patterns across α and AUC values as the above models.

#### Comparison of Cost Savings Across Degrees of Freedom

Figure 4D shows that heterogenous thresholds would increase cost savings by almost US $700 million each year, relative to 80% uniform thresholds and by as much as US $300 million each year, relative to a uniform chosen threshold. These calculations assume an AUC of 0.9 and an α of 20 across the 3 models. An α of 20 aligns with the maximum penalty for false alarms in the PhysioNet challenge [4], and an AUC of 0.9 is close to the predictive accuracy of the latest advancement in sepsis predictive analytics by Shashikumar et al [5].

### DOR: Objective Measure of Accuracy

#### Uniform Classification Threshold

**Figure 5:**
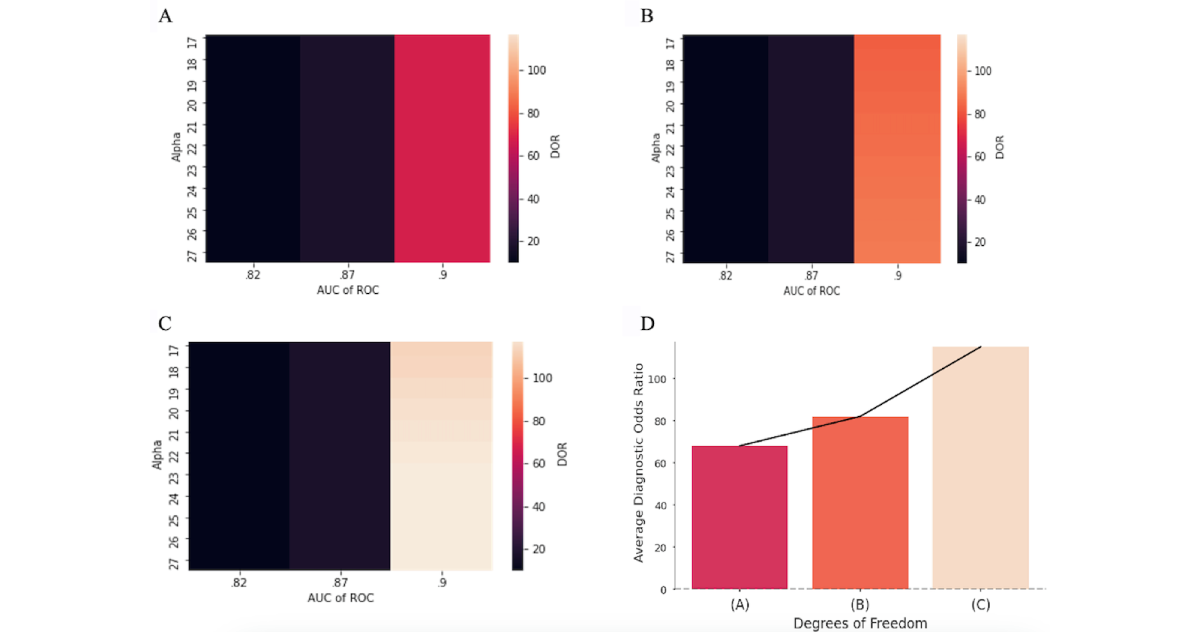
(A) Model diagnostic odds ratio when threshold is 80% sensitivity across departments. (B) Model diagnostic odds ratio when one threshold is chosen, but it is the same across departments. (C) Model diagnostic odds ratio when threshold is chosen across departments. (D) Model diagnostic odds ratio by each model type (A), (B), and (C). The cost savings estimates in (D) are for area under the curve=0.9 and α=20 across the 3 models. We choose α=20 to align with the maximum penalty for false alarms in the PhysioNet challenge [4]. AUC: area under the curve; ROC: receiver operating characteristic curve.

We present an objective measure of accuracy, called the DOR, attained at each AUC-α pair, given the optimal thresholds. Figure 5A illustrates that the highest levels of diagnostic accuracy are achieved when costs are the lowest, suggesting that cost minimization can simultaneously maximize algorithmic performance. Naturally, more predictive models also lead to higher DOR values.

#### Uniform Classification Threshold Chosen by Optimizer

Optimizing the uniform threshold leads to higher DORs at each AUC-α pair. This improvement is prominent in most accurate models, where DOR can differ by as much as 30 between different degrees of freedom (Figure 5B).

#### Heterogenous Classification Thresholds Chosen by Optimizer

Heterogenous thresholds further increase the DOR at every point relative to the previous 2 alternatives. In Figure 5C, we see that DOR reaches up to 116 at the highest point.

#### Comparison of Cost Savings Across Degrees of Freedom

Figure 5D shows that DOR can increase by as much as 50 when switching from a uniform recommended threshold to heterogeneous thresholds, even though DOR is not directly maximized. Interestingly, minimizing excess sepsis costs also leads to higher DOR.

### Savings When Accounting for Provider Adherence

We present results from a set of simulations that fix AUC at 0.87 but which vary the costs of false positives and physician adherence to alarm triggers. Not surprisingly, savings are the highest when adherence is high (see Figure S1 in Multimedia Appendix 1). This result highlights the value of adequate training and quality controls to ensure that physicians and frontline workers who interact with these technologies use them appropriately. Measures to improve physician adherence to alarm triggers could increase cost savings by as much as US $1 billion (US $3 billion at γ=0.5, α=17; US $4 billion at γ=1.0, α=17).

## Discussion

**Figure 6:**
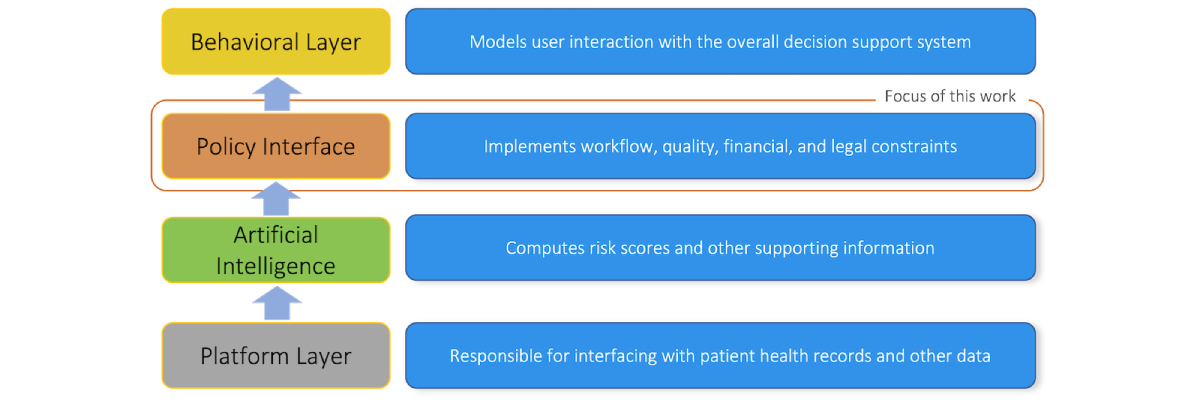
Clinical decision support implementation layers. These layers (and the corresponding key attributes) include the (1) platform layer (interoperability, scalability, and fault-tolerant), (2) artificial intelligence layer (accuracy, generalizability, and interoperability), (3) policy layer (specific and applicable to local hospital workflows, optimality with respect to enterprise’s objectives), and (4) behavioral layer (usability, compliance).

This work estimates the national excess costs of sepsis to CMS and provides a framework for implementing predictive models in clinical settings. Our framework focuses on the policy interface layer of ML implementation, as shown in Figure 6, and chooses classification thresholds or the points above which a patient is deemed septic across broad diagnostic categories to minimize the costs of undertreatment and overtreatment. We illustrate that implementing such algorithms nationwide could potentially save the CMS over US $4.6 billion each year from inpatient hospital-related costs alone. As much as 12.3% of these savings are attributable to our framework for implementation alone, relative to adhering to uniform classification thresholds. We find that diagnostic accuracy would also improve by as much as 68%.

Our work expands the frontier of research on clinical predictive models in several directions. First, we provide a methodology for calculating the excess costs of a given condition and apply that method to sepsis care. Second, to our knowledge, we are the first to provide a framework for optimizing the parameters of predictive models according to the patient subpopulation. Third, our framework is the first to explicitly balance the costs of undertreatment (ie, false negatives) and overtreatment (ie, false positives) by using a constrained optimization routine. Fourth, we allow for a flexible set of hospital-specific parameters that can be rationalized and set by the implementer. Among these, we include the possibility of imperfect adherence to triggered alarms (ie, behavioral failures) or other factors that might influence the effectiveness of the sepsis treatments, given the alarm is followed (imperfect treatment). We also include a flexible parameter identifying the costs of false positives (ie, overtreatment). Since this cost is a difficult value to ascertain and is specific to a given hospital and condition, we allow the user to set this parameter at a level that they deem reasonable. We show that across various assumptions on physician adherence and overtreatment costs, our framework can dramatically increase excess cost savings.

By contrast, recent work in predictive analytics considers the cost of prediction in terms of the number of laboratory tests and their associated costs [32]. However, these approaches overlook the more significant costs incurred from avoidable hospital expenses and insurance payouts that could be prevented by more timely and appropriate health care. Our implementation directly minimizes these costs to optimize predictive analytics. Our approach also allows hospitals and practitioners to reap savings under current DRG-based payment models and value-based care systems [33]. For example, under the increasingly used model of capitation payments, hospitals are allotted a payment for a fixed number of patient lives. Our implementation allows hospitals to optimize their predictive analytics within patient subgroups and provide targeted treatment depending on the needs of those subgroups. Excess cost savings from this targeted approach would be directly reaped by hospitals, incentivizing the adoption of new prediction technologies.

A comparison of results across different model parameter values and inclusion criteria offers several broader insights. First, improving provider compliance to algorithmic recommendations can yield substantial cost savings. These savings are as large as those reaped when setting classification thresholds by broad diagnostic categories, highlighting the importance of dedicating time and resources to the behavioral layer (see Figure 5) of the clinical decision support process. By improving compliance to algorithmic recommendations and optimizing model parameters by patient subpopulation, costs can be further reduced by as much as 40%. Thus, the value proposition of new predictive models depends on how well algorithms are implemented.

Second, our model could be extended to allow provider compliance rates that vary by department. These heterogenous compliance rates could, in turn, affect cost-savings outcomes. Further, one could simulate the potential savings of educational interventions that improve compliance rates within low-compliance departments.

Third, broadening the inclusion criteria of these technologies may lead to much higher excess cost savings. Our strategy, for example, only includes patients with ICD codes corresponding to severe sepsis and septic shock. By contrast, if the inclusion criteria were expanded to cover patients with any sepsis ICD code [34] that maps to the sepsis DRG codes (ie, 870-872), the excess cost savings could double (see Multimedia Appendix 1). Moreover, if predictive technologies were deployed beyond inpatient settings such as in outpatient clinics, skilled nursing facilities, or via at-home wearable devices [35], cost savings could further increase.

Lastly, the cost of false alarms can greatly affect the potential for cost savings. If the costs of sepsis overtreatment are high relative to the costs of undertreatment (eg, worst-case antimicrobial resistance scenarios), cost savings are limited. Identifying these costs, thus, is critical to identifying optimal classification thresholds. However, these costs could vary by hospital or department and may merit more specific calculations.

Our analysis, of course, has limitations. First, it is difficult to estimate the true excess costs of sepsis. Our estimates, which compare patients within the same DRG and with similar baseline comorbidity indices, attempt to isolate the effect of sepsis on excess costs. Our estimates, however, are an imperfect attempt at identifying the causal effect of sepsis on costs and could include other factors that increase costs apart from sepsis. Second, our analysis uses data from only one hospital. Obtaining fine-grained costs from hospitals is an arduous process; thus, we are limited by our sample size. Further, we do not account for the value of lives saved from improved treatment and any costs incurred after discharge, despite readmissions from sepsis being extremely common and expensive [36]. Thus, we analyzed the excess costs of patients with sepsis for whom sepsis is a nonprimary diagnosis while accounting for other primary reasons for hospital admission. This allowed us to analyze avoidable costs that could be prevented by early sepsis detection during hospital care. Third, there are other factors not discussed here that could push providers to not respond to alerts (eg, wrong person alerted, outdated data, repeated alerts). Finally, a patient’s MDC category is often not known until discharge, which may complicate prospective patient-level analysis. However, our proposal is focused on driving department-level policies from retrospective data for the implementation of predictive analytic algorithms. Further, recent work has demonstrated the feasibility of predicting DRG codes in real time [37,38]. Despite these limitations, we believe that our analysis serves as a useful framework for the deployment of predictive analytics in clinical settings and underscores the potential savings when these models are deployed in a manner that directly considers costs.

We show that fine-tuning prediction technologies to perform well under behavioral and cost constraints can improve patient outcomes while reducing health care spending. We estimate that CMS could save over US $4.6 billion each year from inpatient hospital-related costs alone and that diagnostic accuracy would improve by as much as 68% through the use of an ML algorithm to predict sepsis. Our results suggest that the value proposition of new prediction technologies can be improved through fine-tuning within a clinical setting. Prospective studies are needed to validate these findings.

## Appendix

Multimedia Appendix 1 Supplementary material.

## Abbreviations

AUC: area under the curve
CMS: Centers for Medicare and Medicaid Services
DOR: diagnostic odds ratio
DRG: diagnosis-related group
ICD: International Classification of Diseases
MDC: major diagnostic category
ML: machine learning
STROBE: Strengthening the Reporting of Observational Studies in Epidemiology
UCSDH: University of California San Diego Health

## Data Availability

The financial data underlying this study cannot be shared publicly due to the proprietary nature of the data. Centers for Medicare Provider Utilization and Payment Data (inpatient) used for the extrapolation of costs and case-mix adjustments can be found at [30] and [39]. The supplementary material in Multimedia Appendix 1 is available in the Journal of the American Medical Informatics Association online.
