# Supplementary material for: Optimizing the Implementation of Clinical Predictive Models to Minimize National Costs: Sepsis Case Study
Source: J Med Internet Res. 2023 Feb 13;25:e43486. doi: 10.2196/43486 (PMC9972209; doi:10.2196/43486)
Supplement: Multimedia Appendix 1 [file jmir_v25i1e43486_app1.docx]

**SUPPLEMENTARY MATERIAL**

1. *Cost Minimization Routine Across Diagnostic Categories*

We choose the sensitivity of the algorithm in each MDC code *i* to minimize sepsis payments subject to boundary constraints. The primary constraint is the tradeoff between sensitivity and specificity: higher sensitivity leads to lower specificity. The sharpness of that tradeoff is determined by the ROC curve. The formulation of the optimization routine is as follows:


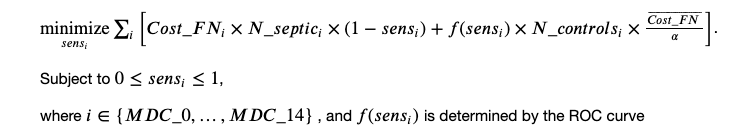


**Equation S1**. Main minimization routine.

Here, *sens_i_* is sensitivity and *spec_i_* is specificity. *Cost_FN_i_* is given by the cost of false negatives in the given MDC code *i*. This was gathered earlier by calculating the difference between CMS payments for patients with sepsis within a given DRG weight (DRG and quarter) and patients without sepsis within that same DRG weight that were matched on baseline CCI (severity). *N_septic_i_* is the number of septic patients in the given MDC code and (*1 – sens_i_*) is the miss rate. Thus, the left-hand side of the objective function can be thought of as the total additional payments made by CMS for septic patients. *f(sens_i_)* is the false positive rate, which is calculated using a fitted function *f( )* to the predictive model’s ROC curve. The estimated functional form *f( )* thus provides a mapping from the chosen sensitivity to specificity. *N_controls_i_* represents the total number of patients in the MDC code/department that do not have sepsis. The parameter *Cost_FN* bar is the average cost of false negatives across all MDC codes. The parameter *α* is a variable that maps the cost of false positives to the cost of false negatives as there may be a cost of giving someone antibiotics if they do not have sepsis (e.g., adverse side effects). Thus, the right-hand side of the objective function can be thought of as the additional cost from false positives.

1. *Cost Minimization Routine with Imperfect Adherence*

To model the cost effects of imperfect physician adherence to alarm triggers, we reformulate our model to allow a portion of true positives to be ignored (behavioral failure). Alternatively, our model extension could account for a treatment failing to prevent sepsis even if the alarm is followed (treatment failure). To this end, we add a *γ* exponent to the miss rate to scale up the miss rate for a given choice of sensitivity:


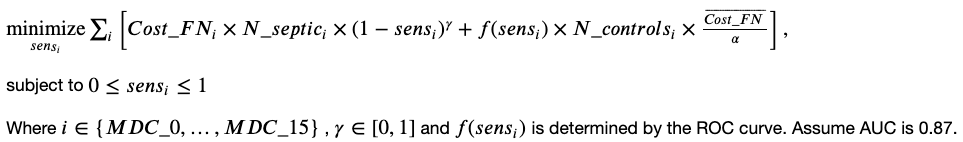
**Equation S2**. Minimization Routine with Imperfect Adherence to Alarm Triggers.

For this model, we use the model that has an AUC of 0.87. Note that the $\gamma$exponent is between 0 and 1 and the miss rate is between 0 and 1. Thus, as $\gamma$decreases, the miss rate increases.


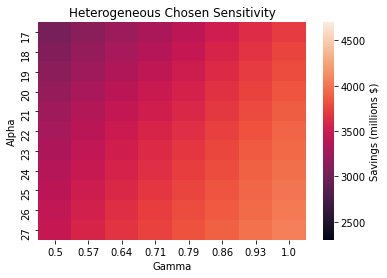


**Figure S1**. Cost Savings Across α Values and Physician Adherence Levels.

1. *Cost Minimization Routine with Uniform Sensitivity Across All MDCs*

Now we compare having the flexibility to determine sensitivity/specificity of the algorithm across departments (MDC codes) to simply choosing one uniform sensitivity/specificity pair across all MDCs.

The formulation for this optimization routine is as follows:


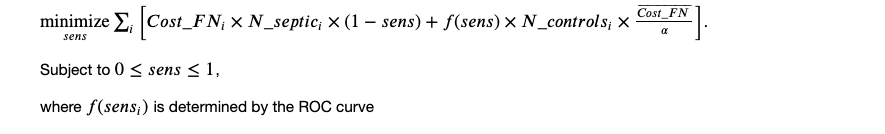
Notice that, instead of choosing a set of sensitivity values across diagnostic categories, we choose only one sensitivity value across the entire hospital.

1. *Cost Minimization Routine With 80% Uniform Sensitivity Across all MDCs*

For this simulation we simply impose a uniform 80% sensitivity across all diagnostic categories consistent with uniform, recommended levels. In this context, no algorithmic optimization for a sensitivity value is used. Costs are calculated by summing up the costs across diagnostic categories with the uniform 80% sensitivity value and the corresponding specificity value determined by the ROC curve.

1. *ROC Curve Smoothing for AUC Constraint*

To fit a function to the ROC curve, to bound the accuracy of the model, we first invert the ROC curve data points to get a mapping between *y = f(sens)*, where y is the false-positive rate, which we insert directly in the objective function. We then transform sensitivity and specificity pairs from the actual ROC curve into logit space using the following transformation


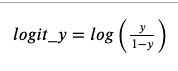


While in logit space we then fitted a model that mapped sensitivity (true-positive rate) to the false positive rate using the following regression model:


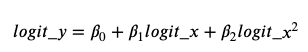


Once we obtain the fitted coefficients, we mapped each value of sensitivity to the false-positive rate by first transforming sensitivity into logit space, generating the logit false positive rate, then returning the logit false-positive rate back into the normal false-positive rate through the inverse logit function.

Using interpolation methods to estimate the ROC curve generated functions that were not smooth and that often lead the optimizer to settle on unstable solutions. With our method, we guarantee a smooth, monotonic function for every value of sensitivity.

Below we plot the original data for the ROC curve vs. the fitted data:


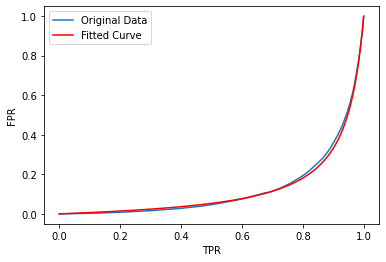


1. *Scaling Excess Costs at UCSDH To National Level*


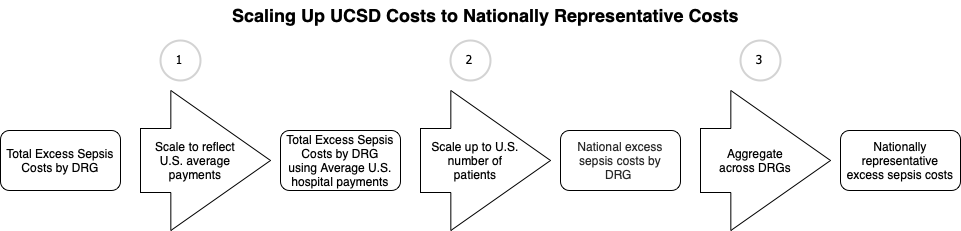


(1) To adjust for payment differences between the average U.S. hospital and UCSDH, we calculate the ratio of UCSDH average payments to US average payments by DRG and scale costs by this ratio

(2) To scale excess costs to a national level, we calculate the share of US patients treated at UCSDH by DRG and divide by this share

(3) Aggregate costs across DRGs

Scholars have valued the total U.S. annual costs of sepsis among Medicare Beneficiaries at $41.8B^19^. This number is derived estimating the costs from patients within the sepsis DRGs 870-872. There are two potential shortcomings of this estimate. First, it does not include sepsis diagnoses that occur in other DRG codes. By only analyzing these patients, some costs could be missed. Second, these costs are not net of the counterfactual level of care these patients would have received without sepsis. It is plausible that even if these patients did not have sepsis as a primary reason for admission, that these patients would have still been admitted to the hospital for other reasons in the near term (I.e., since these patients have more underlying conditions). Measuring the counterfactual level of costs, a patient would have incurred is essential for determining the actual cost of sepsis. That is, if we could prevent sepsis from occurring, what savings could society reap?

By analyzing patients outside the sepsis DRGs, we can do comparisons between individuals with similar underlying conditions and primary reasons for admission, but where sepsis is the key differentiator. Thus, our estimates provide a reasonable estimate of the excess costs of sepsis and can be used to scale the sepsis estimate costs in sepsis DRGs.

To scale our hospital-level estimates of excess costs to the national level, we aggregate all the excess costs of sepsis at the DRG level. Then, we calculate the ratio of UCSDH average payments to US average payments by DRG. This number measures the differences in payments for each DRG that UCSDH receives (e.g., teaching hospital add-on payments). We scale the excess cost totals in each DRG by these ratios to achieve a more nationally representative excess cost measure at the hospital level. Next, to scale these costs up nationally, we calculate the share of national patients that UCSDH treats in each DRG. We then divide the payment-adjusted DRG-level excess costs by these shares to achieve a national DRG excess cost. These costs, however, could be biased by UCSDH’s case mix. Thus, we scale these costs by Medicare’s case-mix index to normalize these costs to represent the national case mix.

The results of this estimation suggest that the excess costs of sepsis in non-sepsis DRGs is $5.2 billion annually. Note that, in our data, there is a similar number of patients within non-sepsis DRGs than within sepsis DRGs. Thus, total sepsis cost estimates may be overestimated by a factor of 10. There are, however, other costs of sepsis not measured in our data. Patients, for example, may receive antibiotic prescriptions from pharmacies, which we do not measure. Assuming we only capture half the costs of sepsis using only inpatient data, this still would amount to a 4-fold overestimation of sepsis costs. However, by including non-sepsis DRGs, the total excess costs may be somewhere close to $10 billion annually ($5 billion in non-sepsis DRGs, scaled by 2 from mismeasurement of non-inpatient costs, and $5 billion in sepsis DRGs).

Further the total number of patients in non-sepsis DRGs with sepsis ICD codes is over three times as large as the number of severe septic patients, which could double the excess cost estimates.

1. *Validation of Scaling Exercise*

To validate our scaling exercise, we focus on total costs incurred in the sepsis DRGs. As a benchmark, we use the Medicare Provider Utilization and Payment Data (Inpatient) the U.S.^29^ (“External Dataset”) and internal costs and Medicare reimbursement data from UCSDH (“Internal Dataset”). Focusing on the latter dataset, we scale the estimates of total costs for sepsis DRGs to the national level (“scaled estimates”), using the same methodology described in the section above. We then compare the total costs of sepsis from the external dataset (i.e., “the true reported values”) to the scaled estimates. We find that these costs are virtually identical. We also find that, among all sepsis patients with DRGs 870-872 (as opposed to severe septic and septic shock group included in our analysis), our scaled sepsis cost estimate using UCSDH healthcare data is the same as the scaled estimate using Medicare cost data for UCSDH. Together, these comparisons reinforce the credibility of our scaling exercise and the accuracy of our UCSDH healthcare data extract.

MDC Table

| **MDC Description** | **Share Septic** | **N Septic** | **Excess**  **Sepsis Costs** | **Optimal**  **Sens./Spec.** |
| --- | --- | --- | --- | --- |
| **Pre-MDC** | 0.07 | 88 | $85739.5 | 0.98/0.75 |
| **Diseases & Disorders of the Nervous System** | 0.04 | 23 | $2451.91 | 0.67/0.97 |
| **Diseases & Disorders of the Ear, Nose, Mouth & Throat** | 0.13 | <5 | $64266.0 | 0.98/0.69 |
| **Diseases & Disorders of the Respiratory System** | 0.04 | 38 | $4883.84 | 0.75/0.96 |
| **Diseases & Disorders of the Circulatory System** | 0.02 | 104 | $29151.76 | 0.89/0.91 |
| **Diseases & Disorders of the Digestive System** | 0.04 | 47 | $32531.72 | 0.93/0.87 |
| **Diseases & Disorders of the Hepatobiliary System & Pancreas** | 0.04 | 28 | $18242.07 | 0.9/0.9 |
| **Diseases & Disorders of the Musculoskeletal System & Connective Tissue** | 0.1 | 23 | $7827.39 | 0.91/0.89 |
| **Diseases & Disorders of the Skin, Subcutaneous Tissue & Breast** | 0.2 | <5 | $7265.0 | 0.94/0.85 |
| **Endocrine, Nutritional & Metabolic Diseases & Disorders** | 0.04 | <5 | $25850.5 | 0.92/0.88 |
| **Diseases & Disorders of the Kidney & Urinary Tract** | 0.02 | 70 | $-23270.1 | N/A |
| **Diseases & Disorders of the Male Reproductive System** | 0.25 | <5 | $765.5 | 0.8/0.94 |
| **Diseases & Disorders of Blood, Blood Forming Organs, Immunologic Disorders** | 0.1 | 6 | $2519.5 | 0.81/0.94 |
| **Myeloproliferative Diseases & Disorders, Poorly Differentiated Neoplasms** | 0.03 | 157 | $-12859.09 | N/A |
| **Infectious & Parasitic Diseases, Systemic or Unspecified Sites** | 0.08 | 12 | $-48800.17 | N/A |
| **Mental Diseases & Disorders** | 0.33 | <5 | $1570.0 | 0.9/0.9 |
| **Injuries, Poisonings & Toxic Effects of Drugs** | 0.05 | 16 | $13975.75 | 0.9/0.9 |
| **Factors Influencing Health Status & Other Contacts with Health Services** | 0.1 | <5 | $103.0 | 0.0/1.0 |
| **Multiple Significant Trauma** | 0.5 | <5 | $46164.33 | 1.0/0.43 |
| **Human Immunodeficiency Virus Infections** | 0.06 | 44 | $-36037.73 | N/A |

**Table S1.** The optimal sensitivity/specificity pairs presented are evaluated at AUC = 0.90 and α = 20 for the heterogeneous chosen model. N/A pairs signify MDC categories for which there were negative excess costs associated with sepsis. This negative excess cost may reflect early death or transfers, or other factors that may correspond to lower CMS payments. We omit these categories from our analysis.

1. *Robustness of Matching Estimator (Extension Using Propensity Score Matching)*

We executed an additional excess cost analysis based on propensity score matching. To this end, for each patient, we predicted the likelihood they received a secondary sepsis diagnosis (since this is the “treatment”) using CCI, gender, and age as predictors. We did not have available data on socioeconomic factors.

Interestingly, excess cost estimates by DRG based on propensity score matching led to higher excess cost estimates than those we present in the main text of our paper. For example, the average Medicare payment difference for septic patients versus non-septic patients in the same DRG was $35,000 when matched on propensity scores (instead of $23,000 when matched just on CCI). Thus, our estimates and simulations in the paper’s main text may be conservative or may better account for underlying factors that drive costs. The histogram of excess cost estimates from the propensity score matching is given below (compare to Figure 2).


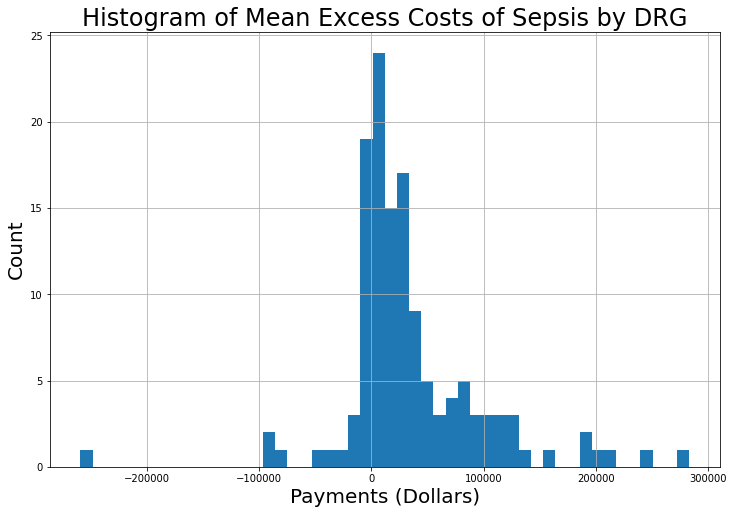


**Figure S2.** Distribution of mean excess sepsis payments over all Diagnosis-Related Groups (DRGs). This is the distribution of excess costs, as presented in Figure 3, but limited to the UCSDH cohort. This figure differs from figure 2 as these cost estimates were generated using propensity score matching.
